# Cis-SNPs Set Testing and PrediXcan Analysis for Gene Expression Data using Linear Mixed Models

**DOI:** 10.1038/s41598-017-15055-8

**Published:** 2017-11-10

**Authors:** Ping Zeng, Ting Wang, Shuiping Huang

**Affiliations:** 1 Xuzhou Medical University, Department of Epidemiology and Biostatistics, Xuzhou, 221004 China; 2 University of Michigan, Department of Biostatistics, Ann Arbor, MI 48104 USA

## Abstract

Understanding the functional mechanism of SNPs identified in GWAS on complex diseases is currently a challenging task. The studies of expression quantitative trait loci (eQTL) have shown that regulatory variants play a crucial role in the function of associated SNPs. Detecting significant genes (called eGenes) in eQTL studies and analyzing the effect sizes of cis-SNPs can offer important implications on the genetic architecture of associated SNPs and interpretations of the molecular basis of diseases. We applied linear mixed models (LMM) to the gene expression level and constructed likelihood ratio tests (LRT) to test for eGene in the Geuvadis data. We identified about 11% genes as eGenes in the Geuvadis data and found some eGenes were enriched in approximately independent linkage disequilibrium (LD) blocks (e.g. MHC). We further performed PrediXcan analysis for seven diseases in the WTCCC data with weights estimated using LMM and identified 64, 5, 21 and 1 significant genes (p < 0.05 after Bonferroni correction) associated with T1D, CD, RA and T2D. We found most of the significant genes of T1D and RA were also located within the MHC region. Our results provide strong evidence that gene expression plays an intermediate role for the associated variants in GWAS.

## Introduction

Since the first study of age-related macular degeneration (AMD) was published in 2005^1^, the past few years have witnessed a remarkably fast development of genome-wide association studies (GWAS)^2^. A large number of genetic susceptibility loci (mostly single nucleotide polymorphisms, [SNPs]) have been identified for many complex diseases^3–6^, including human cancers^7–12^, psychiatric disorders^13–16^, autoimmune-related diseases^17–23^, and many others. However, for most complex diseases, the identified variants only account for a minority of heritable variation, resulting in the so-called missing heritability problem^24^. Additionally, the majority of identified SNPs in GWAS are located within the non-coding regions (e.g. approximately 88% lie in intergenic or intronic regions^4^) and their causal genetic function remains largely unknown. Understanding the functional effects of the non-coding genetic variants is currently one of the main challenges. Recent advances of sequencing technologies have allowed researchers to quickly and cheaply type every genetic variant across the genome. A lot of large scale expression quantitative trait locus (eQTLs) studies^19,25–27^ have been implemented and revealed that many variants identified in GWAS are also regulatory SNPs, which have an important influence on the molecular-level phenotypes (e.g. gene expression)^25,28–31^. This suggests that eQTLs mediate the effects of risk variants in GWAS and hold the fundamental important role to understand the genetic mechanism of disease susceptibility and phenotypic variation^27,32^.

In GWAS literature, linear mixed models (LMM) are one of the most popular approaches, and widely used for multilocus association analysis^33–41^, adjustment for individual relatedness and population stratification^42–44^, genome-wide SNP heritability estimation or heritability partition^45,46^ and genetic prediction^47,48^. LMM is also applied to eQTL studies, including fine mapping^49–51^, predication of gene expression^52–54^ and heritability estimation using cis-SNPs^55,56^. Motivated by the wide flexibility and applicability of LMM and the biologically functional importance of cis-SNPs mentioned above, in the present study based on LMM we develop an efficient likelihood ratio test (LRT) to examine whether a set of cis-SNPs are jointly related to the expression level of the gene that they are located within. We further perform PrediXcan analysis^57^ for seven diseases from Wellcome Trust Case Control Consortium (WTCCC)^17^ by making full use of the estimated effects of cis-SNPs yielded via LMM. We carry out numerical studies to evaluate the power of LRT and adopt the Geuvadis gene expression data^26^ to illustrate our analysis framework.

## Methods

### Overview of linear mixed models

Let **e** be an *n*-vector of continuous phenotype (e.g. gene expression level) measured on *n* independent samples and assume **e** is centered so that we ignore the intercept in the model. Let **X** be an *n* by *q* matrix for *q* covariates, **Z** is an *n* by *p* matrix of genotypes for *p* variants (e.g. cis-SNPs within a predefined gene or other well-defined genetic region). We formulate the relationship between **e**, **X** and **Z** via the following linear mixed model^45,58^

1 $$\begin{array}{rcl}\,\,\,{\bf{e}} & = & {\bf{X}}{\boldsymbol{b}}+{\bf{Z}}{\boldsymbol{\beta }}+{\boldsymbol{\varepsilon }},{\boldsymbol{\varepsilon }} \sim N(0,{{\rm{\sigma }}}^{2}{{\bf{I}}}_{n}),\\ {\beta }_{k} &  \sim  & N(0,{{\rm{\tau }}}^{2}),\end{array}$$

where ***b*** and ***β*** are the effects of covariates and cis-SNPs and are assumed to be fixed and random, respectively; ***ε*** is the *n*-vector of independent and identically distributed residual with variance σ^2^ and **I**_*n*_ is an *n* by *n* identify matrix. In equation (1) the phenotype **e** has marginal mean **X** ***b*** and variance **Σ** = τ^2^ **ZZ**′ + σ^2^ **I**_*n*_ = σ^2^ **V**_λ_ with **V**_λ_ = λ**ZZ**′ + **I**_*n*_ and λ = τ^2^/σ^2^. Note that λ is the signal-noise ratio in equation (1) and is an important quantity related to the SNP-based heritability (denoted as *h* ^2^) by *h* ^2^ = λ/(1 + λ). Efficient estimation algorithms and software (e.g. GCTA^59^) have been designed for large scale applications of LMM to genome-wide genetic data.

### Two applications of LMM

As mentioned above, LMM in equation (1) has widely important applications in genetics, and is the foundation of variance-component based association test, population structure control, phenotypic prediction and heritability estimation. In this paper we are particularly interested in two applications of LMM under the context of gene expression data.

#### Cis-SNPs set association test

The first application of LMM is the cis-SNPs set association test. That is, our objective is to detect whether the *p* cis-SNPs located within a given gene are simultaneously related to the expression level **e** of that gene. Here we only focus on cis-SNPs due to the fact that in terms of previous work most eQTLs are near the regulated gene and only a few eQTLs are trans-acting^49,60,61^ and the effects of trans-SNPs are usually too weak to be detected with a reasonably high power^62^. By treating the effects of cis-SNPs ***β*** as random, the cis-SNPs set association test is equivalent to examining *H*_0_: λ = 0 in equation (1). However, it is a nonstandard hypothesis test in the sense that the parameter of interest λ is on the boundary of the parameter space. Under this situation, the commonly-used asymptotic null chi-square distribution does not necessarily hold^63–67^.

We use likelihood ratio test (LRT) to test *H*_0_: λ = 0 and define the LRT statistic as

2 $$T=2\,{{\rm{\sup }}}_{{\rm{\lambda }}\ge 0}\,[L({\rm{\lambda }})\,-\,L({\rm{\lambda }}=0)],$$

where *L*(λ) is the profile log-likelihood function^68,69^ of equation (1). While the score-based test^34,70^ can be also employed for testing for *H*_0_: λ = 0, we prefer LRT as it has been shown previously that: (i) LRT is more powerful than the score test^40,71^; (ii) in addition to p value for significance test, LRT provides additional useful estimates of unknown parameters (e.g. the estimates of λ and the effects ***β*** of cis-SNPs) for downstream data analyses; while the score test cannot offer such estimates as it only fits the null model (i.e. the simple linear model). The unknown parameter λ is obtained by restricted maximum likelihood estimation (REML)^68,72^ and the exact null distribution of the LRT statistic *T* in equation (2) is obtained via a simulation-based manner (Algorithm 1 in Supporting Information) using the spectral representation^40,73–75^.

In previous work LRT was applied to examine the variance component for multilocus genetic association studies^40^. Although efficient algorithms have been developed^71^, LRT still has a high computational cost because it needs to fit both the null model (i.e. a simple linear model) and the alternative model (i.e. a linear mixed model, fitted using REML via Newton-Raphson iterations). Additionally, the null distribution of the LRT statistic is obtained using a simulation-based algorithm^40,74^ (Algorithm 1 in Supporting Information). Thus, These limit LRT more widespread application to large scale association studies. For genes with relatively large p values (e.g. greater than 0.05), the simulation-based algorithm is fast and needs only a few simulations to yield stable p value estimates. However, it is computationally expensive for genes that have very small p values (e.g. less than 10^−6^). For example, assume there are a total of 20,000 genes, then at least 10^7^ simulations are required to obtain stable p values at the significance level of *α* = 2.5 × 10^−6^ corrected by the Bonferroni method for multiple hypothesis testing, making LRT infeasible for large scale gene-based association studies. Furthermore, for more extremely small p values (e.g. less than 10^−10^), the resulting p value estimates are typically zero due to limited simulations in the simulation-based algorithm, which is less informative for subsequent data analyses. To reduce the computation burden of the simulation-based algorithm and generate more informative p values for these most significant genes, we approximate the exact distribution with an appropriate mixture as previously considered in^76–78^. Specifically, assume the approximate distribution has a mixture form of

3 $$T \sim \phi {{\rm{\chi }}}_{0}^{2}+(1\,-\,\phi )\kappa {{\rm{\chi }}}_{1}^{2},$$

where ${{\rm{\chi }}}_{0}^{2}$ is a point mass at zero and ${{\rm{\chi }}}_{1}^{2}$ is a chi-square distribution with one degree of freedom, *φ* is the proportion parameter and *κ* is the scale parameter. The unknown parameters *φ* and *κ* can be estimated by the method of moment, the quantile regression or the method of local probability^78^. The corresponding p value of *T* is yielded from the estimated approximate distribution of equation (3) (Algorithm 2 in Supporting Information).

#### PrediXcan analysis based on BLUE

Once λ in equation (1) is estimated by REML, say $\hat{{\rm{\lambda }}}$, we obtain the best linear unbiased estimator (BLUE) for the random effects of the cis-SNPs

4 $$\hat{{\boldsymbol{\beta }}}=\hat{{\rm{\lambda }}}{{\bf{Z}}}^{T}{(\hat{{\rm{\lambda }}}{\bf{Z}}{{\bf{Z}}}^{T}+{{\bf{I}}}_{n})}^{-1}({\bf{e}}-{\bf{X}}{({{\bf{X}}}^{T}{\hat{{\bf{V}}}}_{\hat{{\rm{\lambda }}}}^{-1}{\bf{X}})}^{-1}{{\bf{X}}}^{T}{\hat{{\bf{V}}}}_{\hat{{\rm{\lambda }}}}^{-1}{\bf{e}}).$$

The BLUE $\hat{{\boldsymbol{\beta }}}$ can be employed for genetic prediction, called the best linear unbiased prediction (BLUP)^79^, in both GWAS^47,48^ and gene expression data^54^. Here we apply $\hat{{\boldsymbol{\beta }}}$ as weights in the recently developed PrediXcan analysis^57^ for gene-based test in transcriptome-wide association studies^80^. Specifically, let **G** be the same set of cis-SNPs as **Z** for a given gene and **y** be the phenotype in the GWAS. The basic idea of PrediXcan analysis is first to impute the unobserved gene expression level using weights (e.g. $\hat{{\boldsymbol{\beta }}}$ in equation (4)) estimated from a reference transcriptome data^57^, say, ${\hat{{\bf{e}}}}_{{\rm{gwas}}}={\bf{G}}\hat{{\boldsymbol{\beta }}}$. Note that, here we explicitly adopt the subscript “gwas” to emphasize that we are predicting the unmeasured gene expression in the given GWAS data with **G** and $\hat{{\boldsymbol{\beta }}}$ rather than predicting gene expression with **Z** and $\hat{{\boldsymbol{\beta }}}$. Then, we test for the relationship between **y** and ${\hat{{\bf{e}}}}_{{\rm{gwas}}}$ via a linear model or logistic model depending on **y** is a continuous or binary (e.g. case-control) phenotype. Of note, to generate weights in the PrediXcan analysis, the elastic net (ENET)^57^ and Bayesian sparse linear mixed model (BSLMM)^80^ were also used previously. We will compare the performance of various weights (generated from LMM, ENET and BSLMM) in our real data applications.

### Numerical Studies

We first evaluated the performance of the approximate LRT (aLRT, based on Algorithm 2) and compared with the exact LRT (eLRT, based on Algorithm 1) on SNPs set testing. To make our numerical studies as real as possible, we selected a region of continuous genotypes **Z** from the Geuvadis data^26^ (see below). The selected genotypes **Z** included 100 cis-SNPs with minor allele frequency (MAF) larger than 0.05 and the sample size *n* was 465. For the type I error control, we randomly selected 10, 25, 50, 75 or 100 markers included into equation (1), and simulated gene expression levels from a standard normal distribution and set the cis-SNPs effect ***β*** to zero. For the statistical power evaluation, we generated ***β*** from a normal distribution with mean zero and varying variances (i.e. τ^2^ = 0.03^2^, 0.08^2^ or 0.10^2^; these values were adopted to ensure a reasonable power); again we randomly selected 10, 25, 50, 75 or 100 markers included into equation (1), and simulated gene expression levels from a normal distribution with mean **Z** ***β*** and variance 1. We set *M* to 10^6^ in Algorithm 1 and *L* to 10^4^, 5 × 10^3^, 10^3^ or 500 in Algorithm 2. Here *M* and *L* are respectively the number of simulations used in Algorithm 1 and Algorithm 2 in Supporting Information. The number of replicates was 10^6^ and 10^4^ for the type I error control and statistical power evaluation, respectively. Following previous work^34^, the significance level *α* was set to 10^−4^, and the type I error and power were estimated as the proportion of p values less than *α*.

### Cis-regulatory variants set detection in Geuvadis data

We applied LRT (both aLRT and eLRT) to the Geuvadis data^26^ to perform cis-SNPs set detection. The gene whose expression level is related to at least one cis-SNP is referred to as eGene^81^. Detecting eGene is one of the most important tasks in eQTL studies. Briefly, our aim is to examine whether a set of cis-SNPs that locate within a 10 kb genomic region centered at the transcription start site (TSS) of that gene are related to its gene expression level. These markers are referred to as cis-regulatory variants or cis-expression quantitative trait loci (cis-eQTL) and have important implications for understanding gene regulation and interpreting the genetic basis of variation for complex diseases and traits^4,25,82–85^. In the Geuvadis project^26^ a total of 465 individuals were sequenced on lymphoblastoid cell lines (LCL) from five different populations: CEU, FIN, GBR, TSI and YRI. The genotypes were measured in the 1000 Genomes project. The PEER normalization^26,86–88^ was first used to remove technical variations and then each gene expression measurement was quantile normalized to a standard normal distribution. According to GENCODE^89^ release 12, following^49^ we focused on 15,771 protein coding genes that were expressed on at least half individuals and had at least 10 cis-SNPs, resulting in an average of 75 cis-SNPs (MAF > 0.05) per gene.

### PrediXcan analysis for WTCCC data based on LMM and Geuvadis data

We performed PrediXcan analysis for the Wellcome Trust Case Control Consortium (WTCCC) data^17^. The WTCCC data consists of 2,938 shared controls and about 14,000 cases from seven common diseases: 1,963 individuals with type 1 diabetes (T1D), 1,748 individuals with Crohn’s disease (CD), 1,860 individuals with rheumatoid arthritis (RA), 1,868 individuals with bipolar disorder (BD), 1,924 individuals with type 2 diabetes (T2D), 1,926 individuals with coronary artery disease (CAD), and 1,952 individuals with hypertension (HT). We first imputed missing genotypes of WTCCC using BIMBAM^90^, and further imputed SNPs using the Europe population of 1000 Genomes as the reference panel^91^ with SHAPEIT^92–95^ and IMPUTE2^95^. Finally, we yielded about 2,000,000 SNPs shared across all individuals after stringent quality control (i.e. Hardy-Weinberg equilibrium p value < 10^−4^ and MAF < 0.05). For PrediXcan analysis^57^ we focused on the same 15,771 genes as in the Geuvadis data, and for each pair of genes in the WTCCC and Geuvadis data we matched their cis-SNPs. We predicted the expression level of each gene in the WTCCC data with the weights as the BLUE of cis-SNPs of the corresponding gene in the Geuvadis data, and performed logistic regression for each gene in turn as the WTCCC is a case control study.

### Data availability

Our study did not generate any data and made use of data generated by Wellcome Trust Case Control Consortium. The datasets of WTCCC can be available by application to the Consortium Data Access Committee at https://www.wtccc.org.uk/. The Geuvadis gene expression data can be publicly available at http://www.Geuvadis.org/. The R function implementing LRT (for both aLRT and eLRT) is freely available at https://github.com/biostatpzeng/LRT.

## Results

### Evaluation of type I error and power for numerical studies

Note that evaluating the performance of the approximate LRT (aLRT) is equivalent to evaluating the approximate distribution generated using the mixture method in Algorithm 2. Figure S1 shows the approximate mixture distribution is very consistent to the exact one which is generated using the simulation-based method in Algorithm 1 and has been previously proved to control for the type I error efficiently^40,74^. While we also note that sometimes (e.g. *L* = 500) the approximate distribution tends to be slightly liberal. Table 1 shows aLRT maintains a similar statistical power as eLRT under a range of scenarios. It is seen when the association signal is strong (e.g. τ^2^ = 0.10^2^), aLRT with *L* = 500 generally leads to a slightly higher power than eLRT, corresponding to the finding that the approximate distribution tends to be slightly liberal when *L* is small (e.g. *L* = 500) in Fig. S1. Nevertheless, the inflation of power due to the approximation is acceptable; for example, the greatest difference between the power of aLRT and eLRT is less than 0.017 (Table S1).

**Table 1:** Estimated power for eLRT and aLRT in the numerical studies.

No	eLRT	aLRT			
		10^4^	5 × 10^3^	10^3^	500
**τ** ^2^ = **0.03** ^2^					
10	0.0000	0.0006	0.0006	0.0000	0.0006
25	0.0034	0.0047	0.0050	0.0044	0.0050
50	0.0082	0.0098	0.0095	0.0095	0.0092
75	0.0177	0.0210	0.0210	0.0189	0.0210
**τ** ^2^ = **0.08** ^2^					
100	0.0395	0.0395	0.0395	0.0395	0.0368
10	0.0614	0.0669	0.0675	0.0663	0.0675
25	0.2510	0.2598	0.2595	0.2625	0.2611
50	0.5497	0.5641	0.5625	0.5668	0.5638
75	0.7765	0.7880	0.7880	0.7837	0.7783
**τ** ^2^ = **0.10** ^2^					
100	0.8801	0.8887	0.8881	0.8834	0.8868
10	0.1733	0.1872	0.1850	0.1856	0.1944
25	0.4873	0.4964	0.4987	0.4984	0.5013
50	0.8058	0.8181	0.8194	0.8197	0.8184
75	0.9289	0.9336	0.9349	0.9336	0.9355
100	0.9698	0.9712	0.9712	0.9707	0.9717

Note: The No column denotes the number of cis-SNPs included in the gene; we set *M* to 10^6^ in Algorithm 1 (generate the exact null distribution for eLRT) and *L* to 10^4^, 5 × 10^3^, 1 × 10^3^ and 500 in Algorithm 2 (generate the approximate null distribution for aLRT). The significant level was set to 10^−4^. aLRT: the approximate likelihood ratio test; eLRT: the exact likelihood ratio test.

We further compared the computation time for eLRT and aLRT. A total of 10^3^ genes were tested and each gene included 50 cis-SNPs. The sample size was set to 10^3^. We again set *M* to 10^6^ in Algorithm 1 and *L* to 10^4^, 5 × 10^3^, 10^3^ or 500 in Algorithm 2. The computation was implemented on a personal computer with 3.09 GHz and 3.16 Gb memory and the computation time was averaged over 50 repeats. It shows that eLRT needs about 4.5 hours under this setting, while aLRT needs less than 800 seconds (i.e. about 767, 690, 624 and 616 seconds for *L* = 10^4^, 5 × 10^3^, 10^3^ or 500, respectively), about 20 times faster than the exact counterpart (i.e. eLRT).

### Detection of eGene in the Geuvadis data

**Figure 1:**
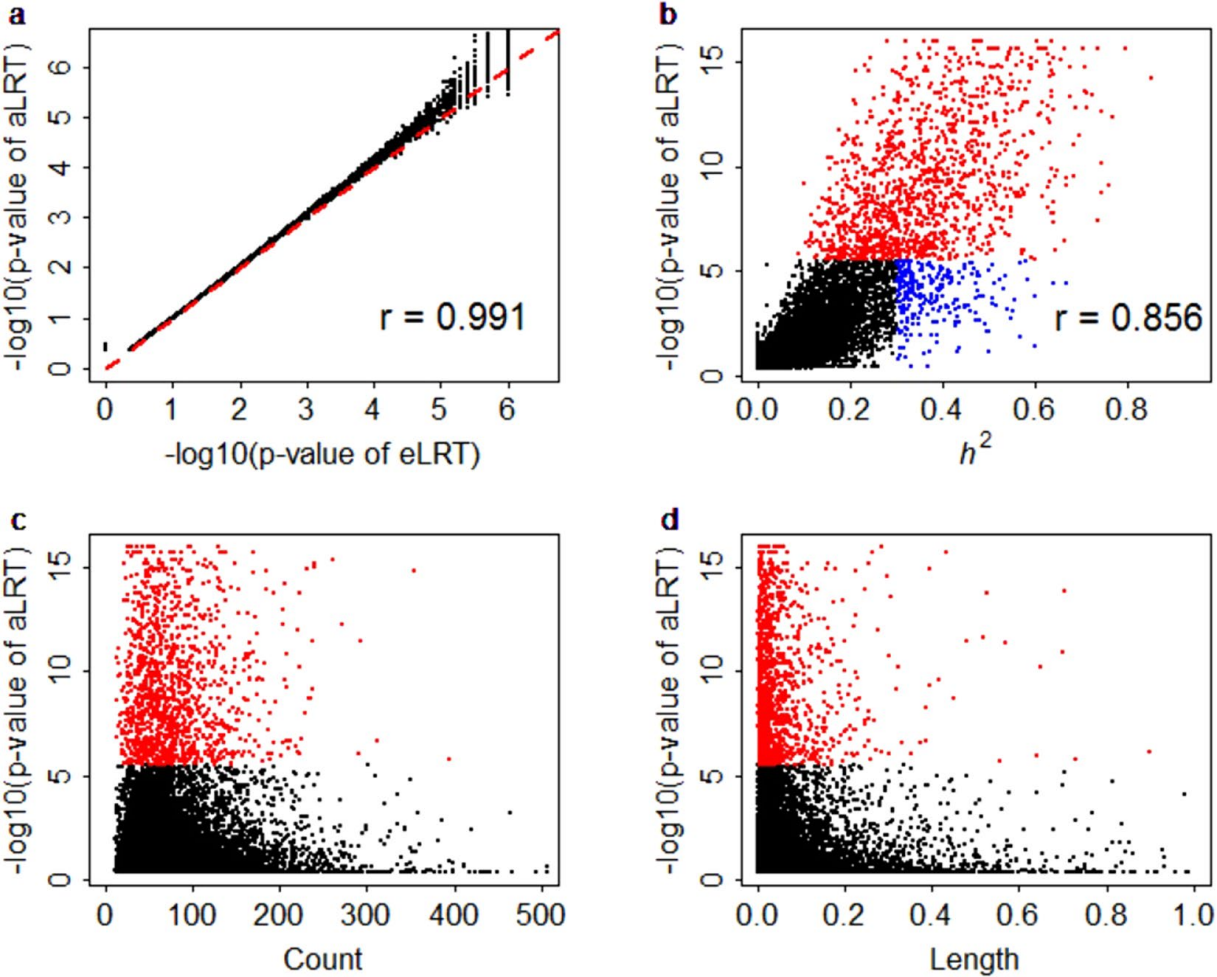
The p values of eLRT and aLRT for all the analyzed genes in the Geuvadis data. (**a**) The scatter plot of p values (with −log10 scale) between eLRT and aLRT across all the genes. (**b**) The scatter plot of p value (with −log10 scale) of aLRT with the estimated heritability of each gene. (**c**) The scatter plot of p values (with −log10 scale) of aLRT with the number of cis-SNPs in each gene. (**d**) The scatter plot of p value (with −log10 scale) of aLRT with the length of cis-SNPs in each gene. eLRT: the exact likelihood ratio test, aLRT: the approximate likelihood ratio test.

Figure 1a displays the p values of aLRT and eLRT. It shows aLRT and eLRT generate comparable results as shown in the numerical studies; the correlation p values (−log10 scale) of aLRT and eLRT is 0.991 (standard error [se] is 8.1 × 10^−4^). We used the Bonferroni method to control for the family wise error rate at 0.05 significance level. After Bonferroni correction, aLRT and eLRT respectively identify 1,665 (10.56%) and 1,707 (10.82%) eGenes. The number of shared eGenes between aLRT and eLRT is 1,653. As a comparison, we also performed the score test, discovering 1,189 eGenes (7.54%), much less than these of aLRT and eLRT. We list the eGenes identified by aLRT but not by eLRT in Table S2, where it shows the p values from eLRT are unstable because of limited simulations (i.e. *M* = 10^6^) in eLRT in Algorithm 1, whereas the p values from aLRT are relatively stable. As mentioned before, it is computationally expensive to obtain believable p values for genes with extremely small p values for eLRT using Algorithm 1; in contrast, aLRT avoids this limitation and offers useful p values, demonstrating the benefit of the approximation strategy. Thereby, the following results are mainly based on aLRT.

**Figure 2:**
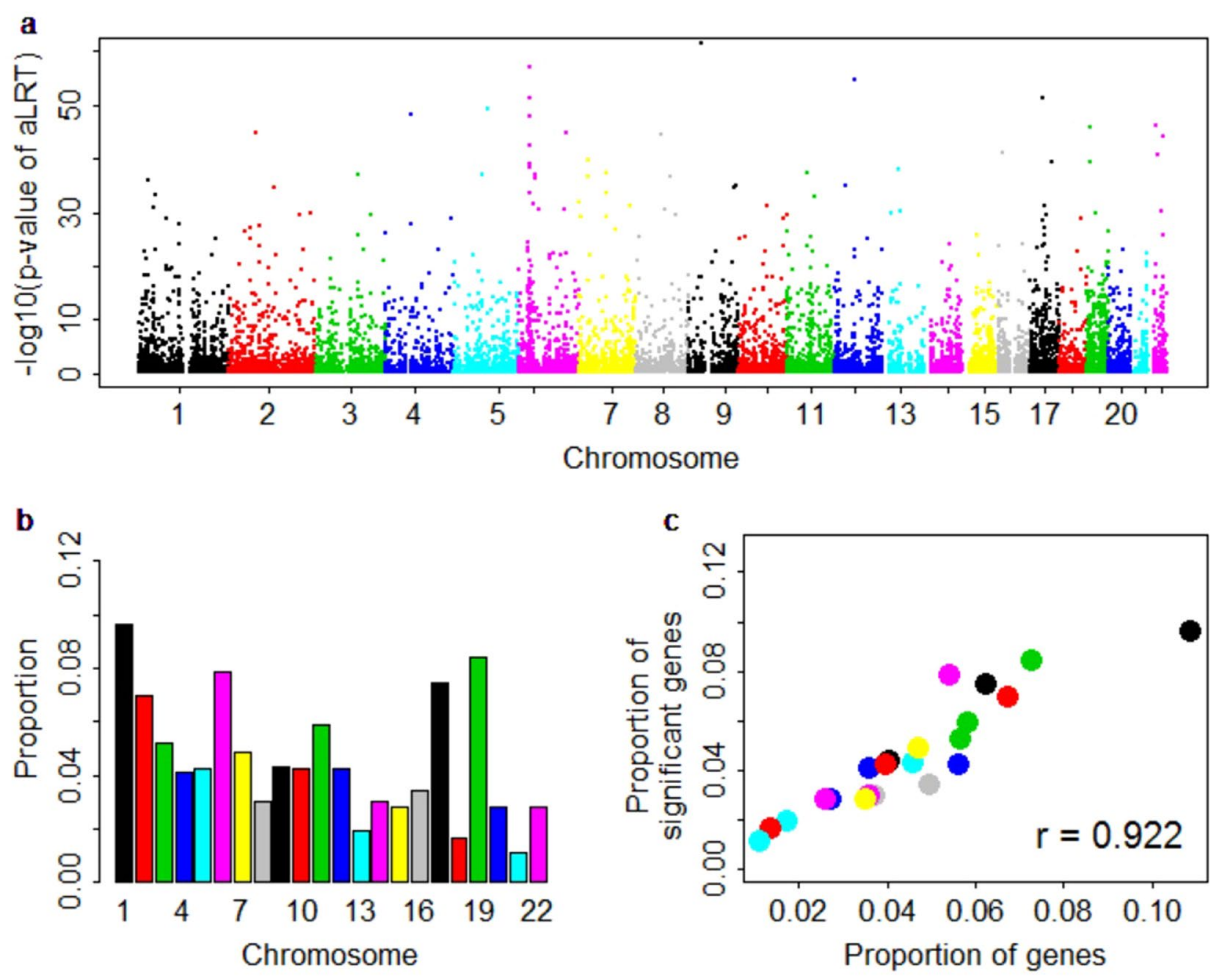
The distribution of p values of aLRT for all genes. (**a**) The Manhattan plot shows p values (with −log10 scale) and gene positions across chromosomes, in which the y-axis is −log10 (p values) for each gene, the x-axis is the gene position and the various colors represent different chromosomes. (**b**) The barplot shows the proportion of significant genes for each chromosome. (**c**) The scatter plot of the proportion of significant genes against the proportion of genes distributed in each chromosome. aLRT: the approximate likelihood ratio test.

To check the distribution pattern of these eGenes, we plot the p values of all genes against the estimated heritability, the number of cis-SNPs included in each gene and the length of the gene in Fig. 1b–d. As expected, it is more likely to be an eGene for a gene with larger heritability (Fig. 1b); the correlation between the p values (−log10 scale) and estimated heritability values is 0.856 (se = 3.2 × 10^−3^). Nevertheless, we do also see that some genes with large heritability fail to be identified as eGenes (e.g. the blue region in Fig. 1b), which may be the direct consequence of the small sample size (i.e. *n* = 465) for the Geuvadis data. We do not see any pattern between the p values (−log10 scale) with the number of cis-SNPs included in each gene (Fig. 1c), and with the length of the gene (Fig. 1d). These observations suggest that a more heritable gene has a higher likelihood to be an eGene, but not all cis-SNPs in a gene have influences on the expression level, and further imply that the genetic architecture of gene expression levels may be less polygenic than that assumed by LMM^54,55,96^. We show the distribution of p values of aLRT for all genes in Fig. 2a, the proportion of eGene for each chromosome in Fig. 2b and the proportion of eGene against the proportion of genes distributed in each chromosome in Fig. 2c. It is seen that chromosomes 1, 2, 6, 11, 17 and 19 include more eGenes, and the proportion of eGene is positively proportional to the proportion of genes of chromosome (the correlation is 0.922 and se = 0.062).

**Figure 3:**
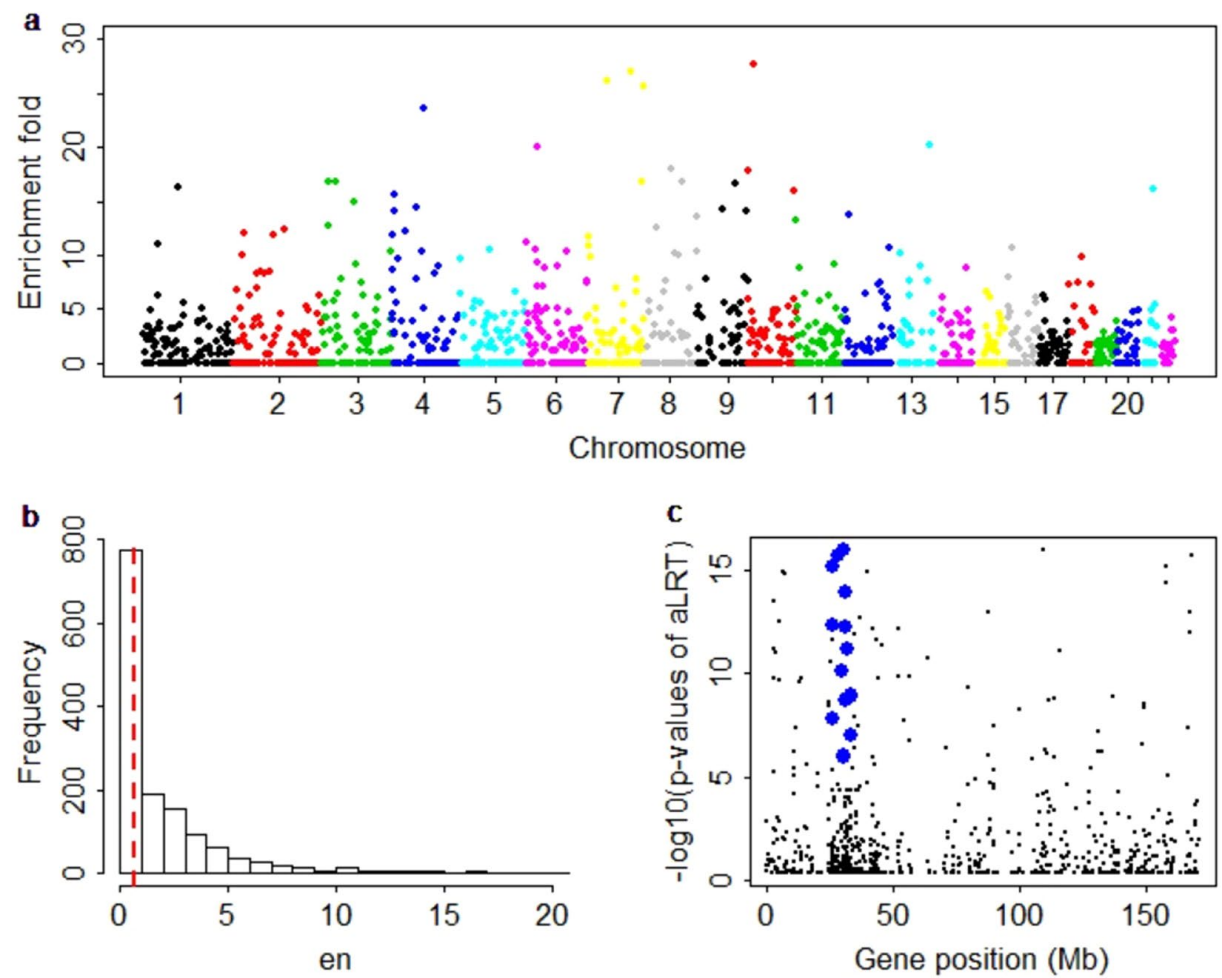
Distribution of enrichment fold for 1,400 approximately independent LD blocks for Geuvadis data. (**a**) A Manhattan-type plot shows enrichment fold for each independent LD block across chromosomes, in which the y-axis is enrichment fold for each LD block, the x-axis is the position of that LD block and the various colors represent different chromosomes. (**b**) The histogram plot shows the distribution of enrichment fold, the median (0.65) of enrichment fold is denoted with a red line. (**c**) The pattern of p values of aLRT (with −log10 scale) for the MHC region (Chr 6: 26–34 Mb). MHC: major histocompatibility complex, T1D: type 1 diabetes, RA: rheumatoid arthritis, LD linkage disequilibrium.

We further examine the enrichment of eGene for approximately independent linkage disequilibrium (LD) blocks across chromosomes. For the Geuvadis data we obtain 1,435 independent LD blocks^97^. We calculate the enrichment fold for each LD block following a similar way as in^98^. In particular, the enrichment fold is computed as the ratio of the proportion of eGene and the proportion of length for the given LD block. We observe enrichments of eGene in some special genetic regions (Fig. 3a) and list these LD blocks with enrichment-fold larger than 20 in Table S3. Here we use the major histocompatibility complex (MHC) region (Chr 6: 26–34 Mb) as an illustrative example. There are 134 eGenes in chromosome 6, among which 36 are located within the MHC region (denoted in blue in Fig. 3c). The total length of chromosome 6 is about 171 Mb, and the length of the MHC region is 8 Mb. Then the enrichment fold is 5.74, which is the ratio of the proportion of eGene in the MHC region (i.e. 0.27 = 36/134) and the proportion of the length of MHC (i.e. 0.05 = 8/171). It is significantly higher (p value is 4.32 × 10^−3^ using an approximate z test) than the average enrichment fold (the median is 1.35) of other LD blocks in chromosome 6. It has long been recognized that the MHC region has importantly biological function on many human diseases and traits^99^. For example, in terms of the NHGRI-EBI GWAS Catalog (http://www.ebi.ac.uk/gwas/, until 05/25/2017), we find that a total of 1,044 (2.72% among all 38,369 variants) identified markers are located within in the MHC region and are associated with as many as about 320 (16.9% among all 1,890 phenotypes) diseases and traits (e.g. type I diabetes, Crohn’s disease, rheumatoid arthritis and infectious diseases)^17,100,101^. However, like most of other identified SNPs, the genetic function of these identified SNPs in the MHC region is also not well understood to date^102^. Therefore, the enrichment of eGene in the MHC region (Fig. 3c) offers a useful understanding for the functional mechanism for these identified SNPs in GWAS.

### PrediXcan analysis results for WTCCC

**Figure 4:**
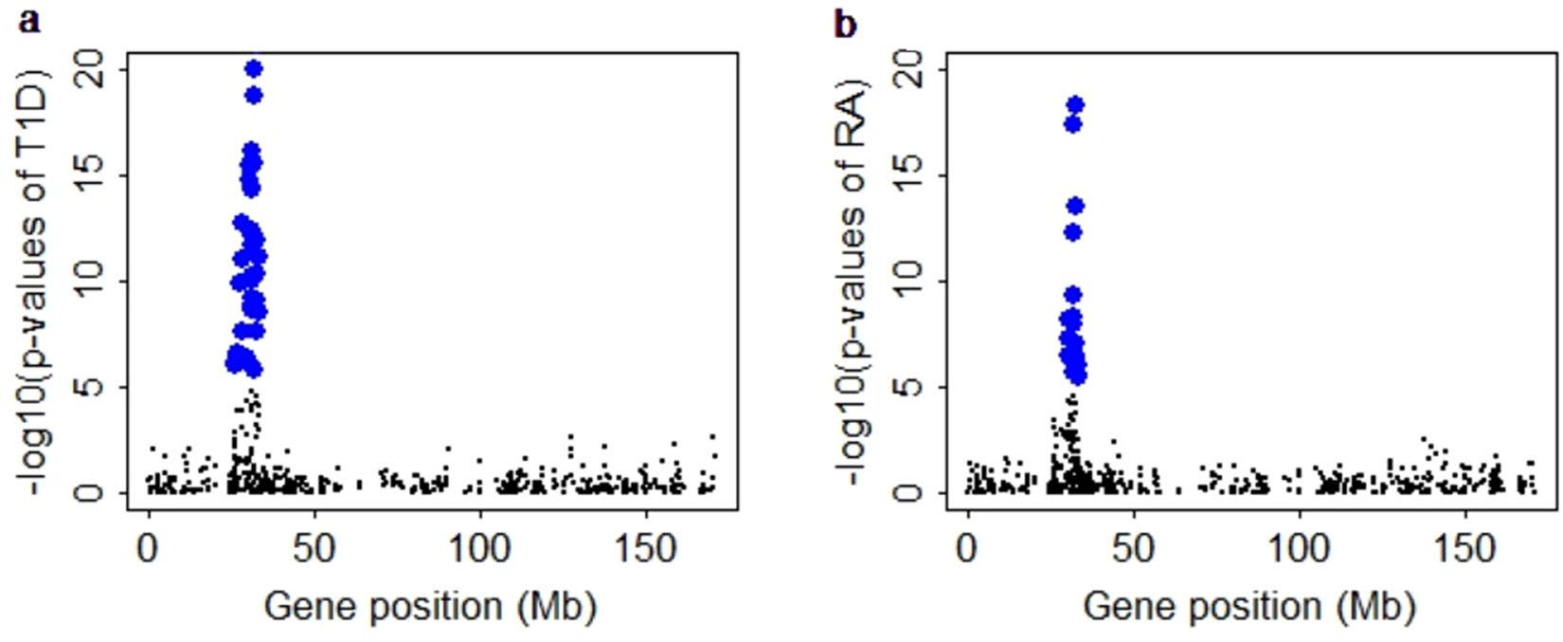
The pattern of p values (with −log10 scale) of PrediXcan analysis of (**a**) T1D and (**b**) RA for chromosome 6. T1D: type 1 diabetes, RA: rheumatoid arthritis.

**Figure 5:**
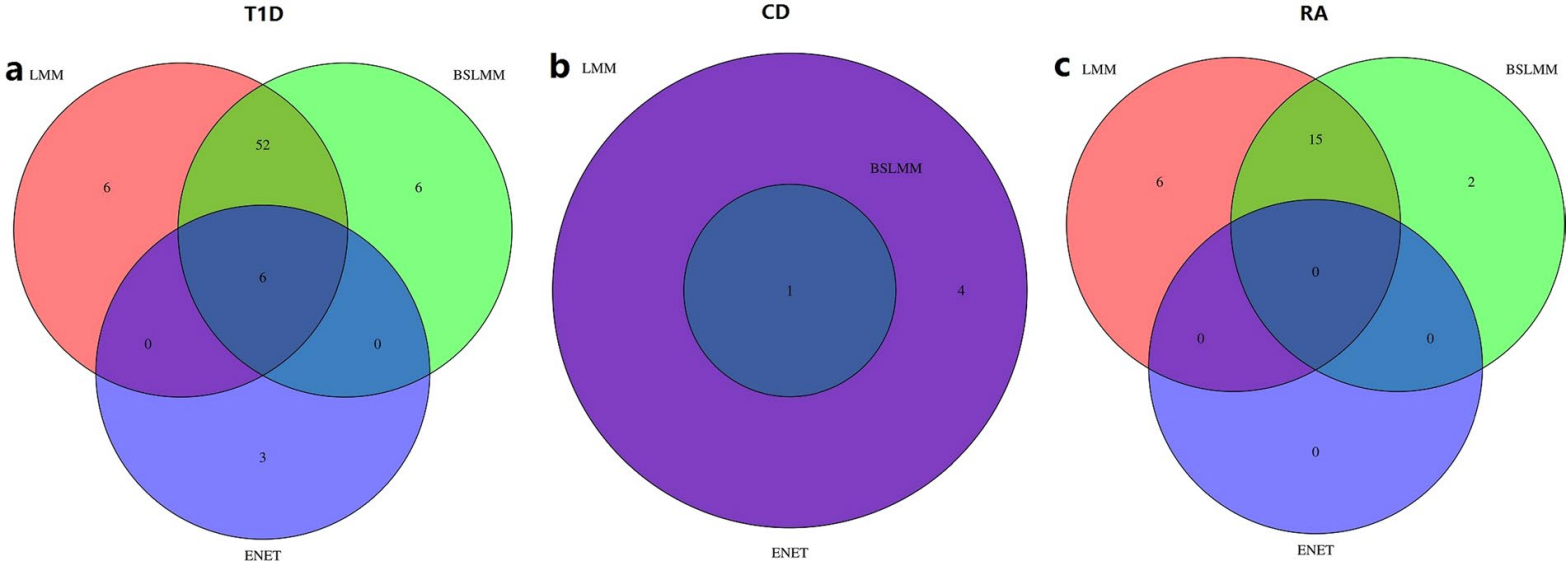
The venn diagram for identified genes of T1D, CD and RA using LMM, ENET and BSLMM. T1D: type 1 diabetes, CD: Crohn’s disease, RA: rheumatoid arthritis, LMM: linear mixed model, ENET: elastic net, BSLMM: Bayesian sparse linear mixed model.

We now turn to the PrediXcan analysis of the seven diseases (i.e. T1D, CD, RA, HT, CAD, BD and T2D) in the WTCCC data. Following^57^ we focus on genes with estimated heritability larger than 0.01, finally resulting in 9,418 genes. Briefly, the BLUE of the cis-SNPs were used to predict the gene expression level using the genotypes of WTCCC; then the predicted gene expression was tested for association with the case-control phenotypes of WTCCC using logistic regression. Manhattan plots summarizing genome-wide association results for the seven diseases are shown in Fig. S2. After Bonferroni correction at 0.05 significance level, 64, 5, 21 and 1 genes are identified that are related to T1D, CD, RA and T2D, respectively. Among these, we observe 57 (89.1%) for T1D and 19 (90.5%) for RA are located within the MHC region (Fig. 4a,b), and all the 76 (57 + 19) genes include risk SNPs that were discovered in previous GWAS (Table S4). Using weights of BSLMM in PrediXcan analysis, 64, 5, 17, 1 and 1 genes are identified that are related to T1D, CD, RA, CAD and T2D, comparable to those identified with LMM; while using weights of ENET in PrediXcan analysis, only 9 and 1 genes are identified that are associated with T1D and CD, much less than those yielded from LMM or BSLMM. Note that the original PrediXcan analysis^57^ based on ENET identified much more significant genes, mainly due to a larger reference data used there^25,57^ — 922 samples were sequenced RNA from whole blood^25^. The venn diagram (Fig. 5) shows the identified genes of T1D, CD and RA are shared among the three methods, especially between LMM and BSLMM. Presumably, the different genes identified with various weights are attributed to the distinct genetic architecture of the gene expression and the diseases as well as the assumptions underlying various models. In summary, together with the enrichments of eGene in the MHC region observed in Fig. 3c in the Geuvadis data, the observations that the significant genes identified by PrediXcan analysis for T1D and RA are also enriched in the same region offer strong supports that gene expression level plays an intermediate role for the risk variants identified in GWAS and the two diseases (i.e. T1D and RA).

## Discussion

In this paper we have applied the popular LMM to the gene expression data. We mainly focus on eGene detection and PrediXcan analysis based on the BLUE of the effects of cis-SNPs. Based on LMM we have employed LRT to discover the eGene in gene expression data, and developed an approximate LRT (aLRT) to speed up the computation. Both numerical studies and real data applications have shown that aLRT works equally well compared with the exact LRT (eLRT) and demonstrated that aLRT can offer more useful estimates for extremely small p values. Importantly, we have shown that aLRT achieves substantial gains in computation while maintaining the effective type I error control and the statistical power. As shown, aLRT is orders of magnitude faster than eLRT depending on the choice of *L*. For example, if *M* = 10^7^ in Algorithm 1 and *L* = 10^3^ in Algorithm 2, theoretically, aLRT can improve the computation approximately 10^4^ times relative to eLRT if ignoring the estimation of the approximate mixture null distribution. For the balance between accuracy and computational cost, in practice we recommend using *L* = 10^4^ since empirically this choice has a higher accuracy compared with smaller values of *L* while not resulting in the increase of the computation burden significantly.

In the Geuvadis data we have shown that eGenes enrich in some special genetic regions (e.g. the MHC region), consistent with the previous finding from a perspective of the prediction of gene expression level^54^. However, we note that the power of eGene detection is still underpowered (e.g. less than 11% in the Geuvadis data with LRT, and less than 8% with the score test) because of the small simple size (i.e. 465 in the Geuvadis data). Incorporating functional annotations of cis-SNPs into the test is a potential way to improve the power^49,81^ and is an active area in eQTL studies. The enrichment of eGenes in some specific genetic regions can offer important implications for SNPs that are identified in GWAS since it is now believed that the function of SNPs on phenotypes works by at least partially regularizing gene expression levels in a cis- or trans-acting manner^27,30,49,61,81^.

Our analysis on two (i.e. T1D and RA) of seven diseases in the WTCCC data has shown that the PrediXcan analysis is an efficient way bridging SNPs, gene expressions and diseases. Especially, the PrediXcan analysis shows the same region (i.e. MHC) of enrichment of significant genes in PrediXcan analysis as that for the eGenes in the gene expression data. This is not likely by chance since there is a lot of evidence that the MHC region has important impacts on T1D and RA^17,100,102^. Nevertheless, we caution that the results of PrediXcan analysis for a given disease may be tissue-specific (e.g. the gene expression of the Geuvadis data used in the present paper was measured from lymphoblastoid cell lines) as it has been shown that the gene expression level is tissue-specific even for biologically developmentally close tissues^27,103–105^. Investigating the performance of PrediXcan analysis on diseases using tissue-specific gene expression level is an interesting problem in the further.

Finally, we recognize that different weights computed using various methods (e.g. LMM, ENET and BSLMM) can be used in PrediXcan analysis^57,80^. Although it has shown the genetic architecture of gene expression is less polygenic compared to most human complex diseases^55,96^ and the sparse LMM has a better performance to capture the variation of gene expression^54^, the optimal weights in PrediXcan analysis is not fully clear and may be case-specific. The property of PrediXcan analysis is also not fully studied and its power relies on many factors, such as the used reference transcriptome data (e.g. the Geuvadis data in the present paper), the genetic architecture of gene expression and the diseases. The weights estimated from LMM may not be the best choice, but in the real applications, we indeed found that the PrediXcan analysis based on LMM behaves comparably relatively to other competing methods. Performing a comprehensive comparison of PrediXcan analysis based on larger reference transcriptome data with various weights on large-scale GWAS phenotypes is our ongoing work.

## Electronic supplementary material

Supplementary Information
