# Supplementary material for: Cis-SNPs Set Testing and PrediXcan Analysis for Gene Expression Data using Linear Mixed Models
Source: Sci Rep. 2017 Nov 10;7:15237. doi: 10.1038/s41598-017-15055-8 (PMC5681585; doi:10.1038/s41598-017-15055-8)
Supplement: Supplementary file 1 — Supplementary Information [file 41598_2017_15055_MOESM1_ESM.doc]

**Cis-SNPs Set Testing and PrediXcan Analysis for Gene Expression Data using Linear Mixed Model**

**Ping Zeng^1,2*^, Ting Wang^1^, Shuiping Huang^1*^**

^1^ Xuzhou Medical University, Department of Epidemiology and Biostatistics, Xuzhou, 221004, China

^2^ University of Michigan, Department of Biostatistics, Ann Arbor, MI, 48104, USA.

* Corresponding authors: [hsp@xzhmu.edu.cn](mailto:hsp@xzhmu.edu.cn ) and zpstat@xzhmu.edu.cn

**Algorithm 1 and Algorithm 2 for the likelihood ratio test**

**Algorithm 1: the exact algorithm based on simulation**. The linear mixed model we consider is

$$\begin{matrix} e = \sum_{j=1}^{q} X_{j}b_{j} + \sum_{k=1}^{p} Z_{k}\beta_{k} + \varepsilon= Xb + Z\beta+ \varepsilon, \\ \beta_{k} \sim N(0, \text{τ}^{2}), \\ \varepsilon\sim N(0, \text{σ}^{2}I_{n}), \end{matrix}$$

where **e** is the gene expression level measured on *n* independent samples, **X** is an *n* by *q* matrix for *q* covariates, **Z** is an *n* by *p* matrix of genotypes for *p* cis-SNPs within a predefined gene. Equation has mean **X*b*** and variance **Σ** = τ^2^**ZZ**′ + σ^2^**I***_n_* = σ^2^**V**_λ_ with **V**_λ_ = λ**ZZ**′ + **I***_n_* and λ = τ^2^/σ^2^. The restricted log-likelihood function for equation is

$L\left(\text{λ}\right) = -\frac{1}{2}\left\{\log|\Sigma| + \log|X^{'}\Sigma^{-1}X| + (e - Xb)^{'}\Sigma^{-1}(e - Xb)\right\} + C$,

where *C* is a constant independent of λ. The LRT statistic is defined as

$T = 2\sup_{\text{λ}\geq0}\left[L(\text{λ}) - L(\text{λ} = 0)\right]$.

The exact finite sample null distribution of the LRT statistic *T* is obtained using the spectral representation[^3-6^](#_ENREF_3)

$f\text{(λ)} = \sup_{\text{λ}\geq0}\left\{(n - q)\log\left[1 + \frac{\Lambda\text{(λ)}}{\Delta\text{(λ)}}\right] - \sum_{j=1}^{p} \log(1 + \text{λ}\mu_{j})\right\}$,

with

$\Lambda\text{(λ)} = \sum_{j=1}^{p} \frac{\text{λ}\mu_{j}}{1 + \text{λ}\mu_{j}}z_{j}^{2}, \Delta\text{(λ)} = \sum_{j=1}^{p} \frac{1}{1+\text{λ}\mu_{j}}z_{j}^{2} + \sum_{s=p+1}^{n-q} z_{s}^{2}$,

where *μ_j_*’s are the eigenvalues of **Z**′**P**_0_**Z** and **P**_0_ = **I***_n_* - **X**(**X**′**X**)^-1^**X**′, and *z_k_*’s are independently standard normal random variables. Then *T* converges to *f*(λ) in distribution[^4-6^](#_ENREF_4). Based on the spectral representation above, it is straightforward to build a simulation-based algorithm to obtain the null distribution[^5^](#_ENREF_5), which is implemented in the R package RLRsim[^7^](#_ENREF_7) (see Algorithm 1 below for details). Let *t_m_* (*m* = 1, 2, …, *M*) be the simulated values of *T* from the simulation-based algorithm, where *M* is the simulation number. The p value of *T* is the proportion that the observed value of *T*, say *t*^0^, is equal to or larger than *t_m_*. We refer to the null distribution consisting of *t_m_* as the exact distribution, and the corresponding LRT as the exact LRT (eLRT).

***Algorithm 1****: Simulation-based algorithm for the exact finite sample null distribution of T*

| step 1. | Compute the eigenvalues *μ*; |
| --- | --- |
| step 2. | Give a grid of candidate values of λ (say 0 = λ_1_ < λ_2_ < … < λ*_N_*), for *m* = 1, 2, …, *M*: |
| (i) | Sample independent standard normal random variable *u_i_*, *i* = 1, 2, …, *n - q*, |
| (ii) | For each λ*_j_* (*j* = 1, 2, …, *N*) compute ∆(λ*_j_*) and *Λ*(λ*_j_*), and obtain $\text{λ}_{\text{max}}^{m}$ that maximizes *f*(λ) given in equation , |
| (iii) | Set $t_{m} = f(\text{λ}_{\text{max}}^{m})$; |
| step 3. | The *p value* of *t*^0^ is computed as: $\sum_{m=1}^{M} I(t_{m} \geq t^{0})/M$. |

**Algorithm 2: the approximate algorithm based on a mixture distribution**. Assume the approximate distribution has the mixture form of

$T \sim\phi\text{χ}_{0}^{2} + (1 - \phi)\kappa\text{χ}_{1}^{2}$,

where $\text{χ}_{0}^{2}$ is a point mass at zero and $\text{χ}_{1}^{2}$ is a chi-square distribution with one degree of freedom, *φ* is the proportion parameter and *κ* is the scale parameter. Analogous mixture distributions were previously considered in[^8-10^](#_ENREF_8). We adopt the method of local probability (MLP) to estimate *φ* and *κ*. Let *t_l_*, *l* = 1, 2, …, *L* (typically, *L* ≤ *M*) be the simulated values of *T* from the exact distribution via Algorithm 1. Since *φ* is essentially the probability of *T* that has a local maximum at λ = 0[^5^](#_ENREF_5), thus we estimate *φ* with the local probability

$$\begin{matrix} \hat{\phi}_{\text{MLP}} =\mathrm{P}\text{rob}\left\{\sum_{j=1}^{p} \mu_{j}u_{j}^{2}<\left(\frac{1}{n-q}\sum_{j=1}^{p} \mu_{j}\right)/\sum_{s=1}^{n-q} u_{s}^{2}\right\}, \\ \hat{\kappa}_{\text{MLP}} =\sum_{l=1}^{L} t_{l}/(1-\hat{\phi}_{\text{MLP}}). \end{matrix}$$

Here $\hat{\phi}_{\text{MLP}}$ is computed by the method of Davies[^11^](#_ENREF_11). These quantities needed for the MLP estimation in equation are by-products of Algorithm 1. One advantage of MLP is that $\hat{\phi}_{\text{MLP}}$ is unchanged regardless of how many simulated values of *t_l_* are used. We summarize the above methods in the following Algorithm 2. We refer to the distribution generated by Algorithm 2 as the approximate distribution, and the corresponding LRT as the approximate LRT (aLRT).

**Algorithm 2: Simulation-based algorithm for the approximate null distribution**

| step 1. | Compute the eigenvalues *μ*; |
| --- | --- |
| step 2. | Give a grid of candidate values for λ (say 0 = λ_1_ < λ_2_ < … < λ*_N_*), for *l* = 1, 2, …, *L*: |
| (i) | Sample independent standard normal random variable *u_i_*, *i* = 1, 2, …, *n-q*, |
| (ii) | For each λ*_j_* (*j* = 1, 2, …, *N*) compute *∆*(λ*_j_*) and *Λ*(λ*_j_*), and obtain $\text{λ}_{\text{max}}^{l}$ that maximizes *f*(λ), |
| (iii) | Set $t_{l}= f(\text{λ}_{\text{max}}^{l})$; |
| step 3. | Use MLP to yield the approximate null distribution; |
| step 4. | Obtain the corresponding p value according to the estimated approximate distribution. |


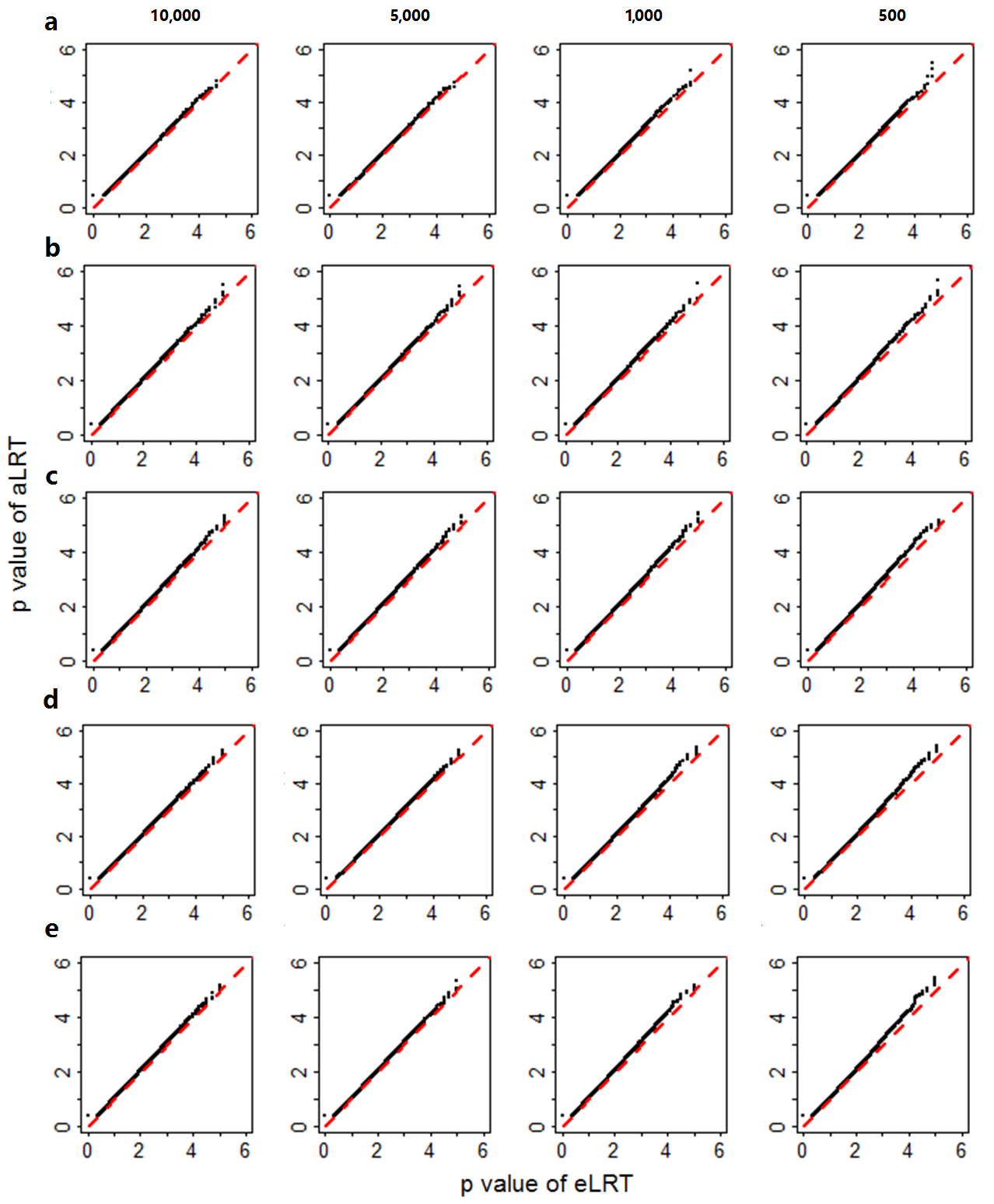


**Figure S1**. **Comparison of the approximate null distribution generated using the mixture method for aLRT with the exact null distribution generated using the simulation-based method for eLRT**. The p values are shown in a -log10 scale. The panel from **a** to **e** respectively corresponds to the simulation setting that includes 10, 25, 50, 75 or 100 markers into the model; and *M* was set to 10^6^ in Algorithm 1 (generate the exact null distribution for eLRT) and *L* to 10^4^, 5 × 10^3^, 1 × 10^3^ and 500 in Algorithm 2 (generate the approximate null distribution for aLRT). aLRT: the approximate likelihood ratio test; eLRT: the exact likelihood ratio test.


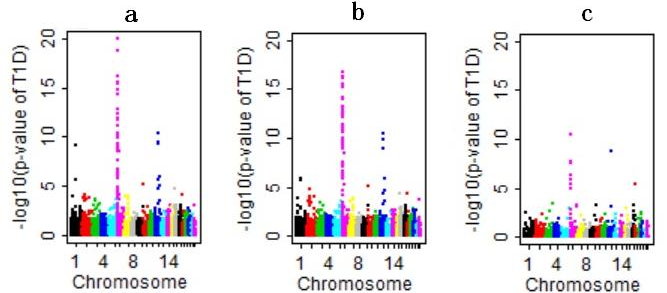


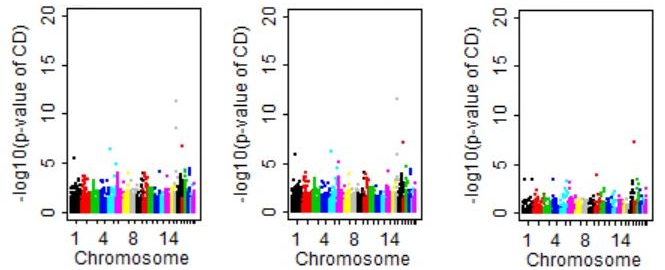


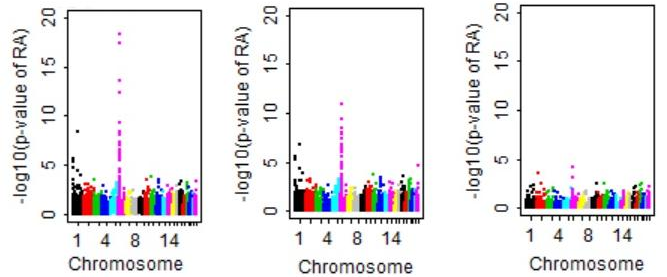


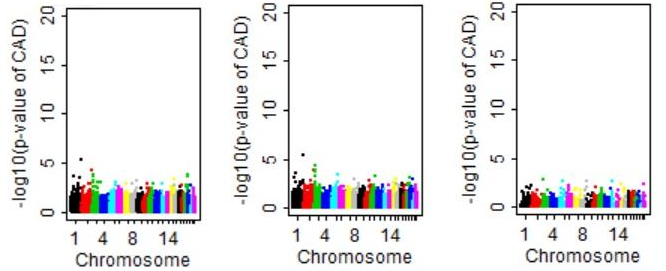


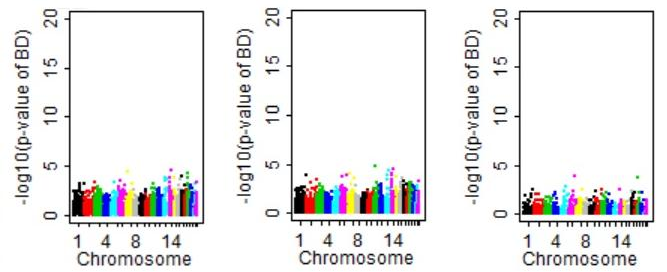


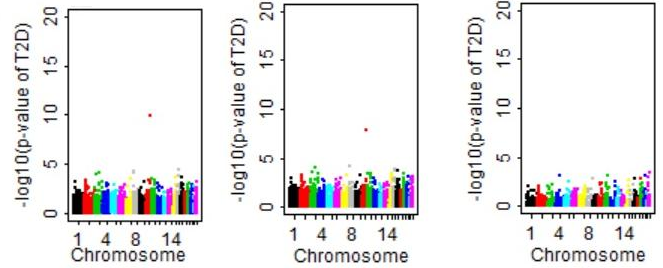


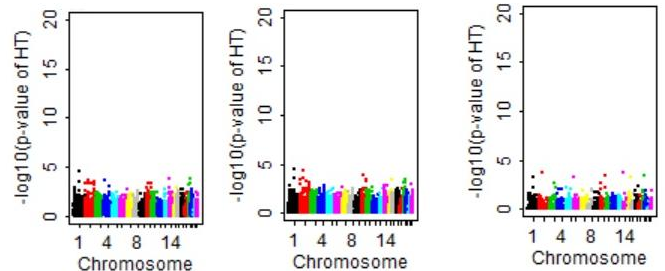


**Figure S2**. **The p values (-log10 scale) of PrediXcan analysis for the seven diseases in the WTCCC data**. (**a**) The p values of PrediXcan analysis using weights estimated via LMM. (**b**) The p values of PrediXcan analysis using weights estimated via BSLMM. (**c**) The p values of PrediXcan analysis using weights estimated via ENET. In each plot various colors correspond to different chromosomes. T1D: type 1 diabetes; CD: Crohn's disease; RA: rheumatoid arthritis; BD: bipolar disorder; T2D: type 2 diabetes; CAD: coronary artery disease; HT: hypertension; LMM: linear mixed model; BSLMM: Bayesian sparse linear mixed model; ENET: elastic net.

**Table S1**. **The power difference between the exact LRT (eLRT) and the approximate LRT (aLRT).**

| No | 10^4^ | 5×10^3^ | 10^3^ | 500 |
| --- | --- | --- | --- | --- |
| τ^2^ = 0.03^2^ |  |  |  |  |
| 10 | 0.001 | 0.001 | 0.000 | 0.001 |
| 25 | 0.001 | 0.002 | 0.001 | 0.002 |
| 50 | 0.002 | 0.001 | 0.001 | 0.001 |
| 75 | 0.003 | 0.003 | 0.001 | 0.003 |
| 100 | 0.000 | 0.000 | 0.000 | -0.003 |
| τ^2^ = 0.08^2^ | | | | |
| 10 | 0.006 | 0.006 | 0.005 | 0.006 |
| 25 | 0.009 | 0.009 | 0.012 | 0.010 |
| 50 | 0.014 | 0.013 | 0.017 | 0.014 |
| 75 | 0.012 | 0.012 | 0.007 | 0.002 |
| 100 | 0.009 | 0.008 | 0.003 | 0.007 |
| τ^2^ = 0.10^2^ | | | | |
| 10 | 0.014 | 0.012 | 0.012 | 0.021 |
| 25 | 0.009 | 0.011 | 0.011 | 0.014 |
| 50 | 0.012 | 0.014 | 0.014 | 0.013 |
| 75 | 0.005 | 0.006 | 0.005 | 0.007 |
| 100 | 0.001 | 0.001 | 0.001 | 0.002 |

**Note**: No in the first column denotes the number of cis-SNPs included in the gene; eLRT: the exact likelihood ratio test, its null distribution was generated in a simulation-based manner (Algorithm 1); aLRT: the approximate likelihood ratio test, its null distribution was generated from the estimated mixture distribution (Algorithm 2); *M* was set to 10^6^ in Algorithm 1 and *L* to 10^4^, 5 × 10^3^, 1 × 10^3^ and 500 in Algorithm 2.

**Table S2. Significant genes identified by the approximate LRT but not by the exact** LRT in the Geuvadis data.

| CHR | Gene | No | Position | eLRT | aLRT |
| --- | --- | --- | --- | --- | --- |
| 1 | *FAAH* | 62 | 46,859,937 | 5.000E-06 | 1.571E-06 |
| 1 | *LRRC8C* | 88 | 90,098,631 | 4.000E-06 | 9.220E-07 |
| 1 | *C1orf198* | 104 | 230,972,865 | 7.000E-06 | 1.787E-06 |
| 1 | *GLUL* | 79 | 182,350,839 | 7.000E-06 | 1.949E-06 |
| 1 | *RP11-350G8.5* | 53 | 154,374,804 | 4.000E-06 | 1.272E-06 |
| 1 | *DENND2D* | 64 | 111,729,796 | 6.000E-06 | 3.017E-06 |
| 1 | *SLAMF7* | 76 | 160,709,037 | 6.000E-06 | 2.121E-06 |
| 1 | *NUF2* | 91 | 163,236,366 | 9.000E-06 | 3.064E-06 |
| 1 | *ADC* | 28 | 33,546,705 | 4.000E-06 | 1.581E-06 |
| 2 | *GGCX* | 57 | 85,774,743 | 8.000E-06 | 1.980E-06 |
| 2 | *POMC* | 41 | 25,383,722 | 8.000E-06 | 2.489E-06 |
| 2 | *KLF11* | 105 | 10,182,976 | 4.000E-06 | 1.466E-06 |
| 2 | *IL1RN* | 88 | 113,864,791 | 5.000E-06 | 1.901E-06 |
| 2 | *GRHL1* | 132 | 10,085,341 | 4.000E-06 | 1.648E-06 |
| 3 | *C3orf52* | 76 | 111,805,182 | 6.000E-06 | 2.812E-06 |
| 3 | *NKIRAS1* | 46 | 23,933,151 | 7.000E-06 | 1.902E-06 |
| 3 | *TMEM45A* | 52 | 100,211,463 | 5.000E-06 | 3.026E-06 |
| 4 | *TLR10* | 61 | 38,773,860 | 7.000E-06 | 3.041E-06 |
| 4 | *ZNF595* | 28 | 53,179 | 6.000E-06 | 2.612E-06 |
| 4 | *PACRGL* | 87 | 20,697,905 | 9.000E-06 | 1.945E-06 |
| 4 | *SPP1* | 63 | 88,896,802 | 4.000E-06 | 2.094E-06 |
| 4 | *LIAS* | 57 | 39,460,620 | 4.000E-06 | 2.794E-06 |
| 4 | *UGDH* | 74 | 39,500,375 | 5.000E-06 | 2.448E-06 |
| 5 | *ZNF354A* | 102 | 178,138,593 | 6.000E-06 | 2.522E-06 |
| 6 | *DTNBP1* | 73 | 15,523,032 | 4.000E-06 | 2.714E-06 |
| 6 | *IER3* | 67 | 30,710,976 | 4.000E-06 | 8.701E-07 |
| 6 | *RP11-367G18.1* | 66 | 113,944,737 | 4.000E-06 | 1.088E-06 |
| 6 | *GNMT* | 24 | 42,928,496 | 5.000E-06 | 2.571E-06 |
| 7 | *FGL2* | 69 | 76,822,688 | 5.000E-06 | 2.500E-06 |
| 8 | *YWHAZ* | 69 | 101,928,753 | 4.000E-06 | 9.862E-07 |
| 8 | *PLEKHF2* | 87 | 96,146,032 | 4.000E-06 | 1.571E-06 |
| 9 | *AK3* | 130 | 4,711,155 | 4.000E-06 | 1.789E-06 |
| 9 | *FAM27E2* | 42 | 45,733,559 | 5.000E-06 | 1.953E-06 |
| 9 | *ALDOB* | 73 | 104,182,860 | 4.000E-06 | 2.127E-06 |
| 10 | *RP11-773D16.1* | 74 | 81,563,813 | 4.000E-06 | 2.365E-06 |
| 10 | *GLRX3* | 104 | 131,934,663 | 4.000E-06 | 2.459E-06 |
| 10 | *C10orf81* | 58 | 115,511,213 | 4.000E-06 | 3.144E-06 |
| 11 | *FIBP* | 42 | 65,651,212 | 5.000E-06 | 3.069E-06 |
| 11 | *SPA17* | 50 | 124,543,694 | 5.000E-06 | 2.973E-06 |
| 13 | *N6AMT2* | 34 | 21,296,543 | 6.000E-06 | 1.254E-06 |
| 13 | *LINC00426* | 109 | 30,914,407 | 4.000E-06 | 2.157E-06 |
| 14 | *COQ6* | 46 | 74,416,629 | 6.000E-06 | 6.487E-07 |
| 16 | *LITAF* | 125 | 11,641,853 | 6.000E-06 | 2.922E-06 |
| 16 | *KAT8* | 21 | 31,127,075 | 5.000E-06 | 2.877E-06 |
| 17 | *FZD2* | 44 | 42,634,827 | 4.000E-06 | 1.442E-06 |
| 17 | *ST6GALNAC2* | 110 | 74,561,461 | 4.000E-06 | 1.666E-06 |
| 17 | *CD300C* | 77 | 72,537,247 | 4.000E-06 | 2.253E-06 |
| 17 | *PSMC5* | 36 | 61,904,810 | 4.000E-06 | 2.486E-06 |
| 17 | *DYNLL2* | 63 | 56,160,780 | 6.000E-06 | 1.868E-06 |
| 18 | *C18orf21* | 24 | 33,552,046 | 6.000E-06 | 2.822E-06 |
| 19 | *HAUS8* | 93 | 17,160,573 | 4.000E-06 | 1.985E-06 |
| 19 | *APOC2* | 76 | 45,449,243 | 5.000E-06 | 2.331E-06 |
| 19 | *ANGPTL6* | 106 | 10,203,013 | 5.000E-06 | 1.137E-06 |
| 22 | *ANKRD54* | 33 | 38,226,862 | 7.000E-06 | 2.334E-06 |

**Note:** The No column is the number of cis-SNPs included in the gene; CHR: chromosome; aLRT: the approximate likelihood ratio test, eLRT: the exact likelihood ratio test.

**Table S3. Independent LD blocks with enrichment fold larger than 20 in the Geuvadis data.**

| Enrichment  fold | # Identified SNPs  in GWAS | # Related diseases  and traits | CHR |  |  |  | LD block | |
| --- | --- | --- | --- | --- | --- | --- | --- | --- |
|  |  |  |  |  |  |  | lower | upper |
| 48.48 | 4 | 3 | 2 |  |  |  | 151,439,708 | 152,195,028 |
| 48.16 | 12 | 8 | 4 |  |  |  | 20,153,655 | 21,928,445 |
| 56.62 | 4 | 4 | 4 |  |  |  | 43,659,802 | 44,883,914 |
| 23.66 | 10 | 10 | 4 |  |  |  | 90,450,587 | 91,779,700 |
| 20.07 | 240 | 120 | 6 |  |  |  | 30,906,147 | 31,679,197 |
| 82.67 | 9 | 8 | 6 |  |  |  | 57,707,424 | 61,938,471 |
| 183.09 | 16 | 12 | 6 |  |  |  | 139,887,129 | 142,330,172 |
| 26.17 | 6 | 6 | 7 |  |  |  | 55,659,078 | 57,386,580 |
| 26.95 | 30 | 14 | 7 |  |  |  | 120,832,509 | 121,720,866 |
| 25.65 | 22 | 16 | 7 |  |  |  | 153,304,952 | 154,964,730 |
| 73.55 | 3 | 3 | 8 |  |  |  | 84,388,144 | 86,069,241 |
| 27.63 | 9 | 9 | 10 |  |  |  | 18,577,273 | 19,756,884 |
| 20.13 | 11 | 11 | 13 |  |  |  | 101,098,524 | 102,864,711 |
| 47.59 | 21 | 20 | 15 |  |  |  | 86,170,947 | 88,276,555 |
| 69.26 | 43 | 26 | 16 |  |  |  | 82,034,709 | 83,003,384 |
| 49.95 | 10 | 9 | 18 |  |  |  | 61,614,382 | 62,403,781 |

**Note:** We obtained a total of 38,369 genetic variants identified in GWAS from <http://www.ebi.ac.uk/gwas/> (until 05/25/2017). We counted the number (the second column) of identified SNPs and the related diseases and traits (the third column) within each LD block. LD: linkage disequilibrium; CHR: chromosome.

**Table S4. Significant genes of PrediXcan analyses for T1D and RA in the MHC region.**

| Diseases | Gene | Position | | *h*^2^ | Selected References |
| --- | --- | --- | --- | --- | --- |
|  |  | lower | up |  |  |
| T1D | *BTN3A2* | 26,365,387 | 26,378,546 | 0.620 | [12](#_ENREF_12) |
| T1D | *BTN3A3* | 26,440,700 | 26,453,643 | 0.262 | [12](#_ENREF_12) |
| T1D | *RP11-457M11.5* | 26,686,469 | 26,688,192 | 0.672 | [12](#_ENREF_12) |
| T1D | *LINC00240* | 26,988,232 | 26,991,703 | 0.255 | [12](#_ENREF_12) |
| T1D | *HIST1H2BO* | 27,861,203 | 27,861,669 | 0.031 | [12](#_ENREF_12) |
| T1D | *ZNF165* | 28,048,753 | 28,057,341 | 0.231 | [12](#_ENREF_12) |
| T1D | *ZNF192* | 28,109,716 | 28,124,089 | 0.041 | [12](#_ENREF_12) |
| T1D | *ZNF193* | 28,192,664 | 28,201,260 | 0.209 | [12](#_ENREF_12) |
| T1D | *NKAPL* | 28,227,098 | 28,228,736 | 0.028 | [12](#_ENREF_12) |
| T1D | *HLA-F* | 29,690,552 | 29,706,305 | 0.390 | [12](#_ENREF_12) |
| T1D | *TUBB* | 30,687,978 | 30,693,203 | 0.013 | [13-17](#_ENREF_13) |
| T1D | *FLOT1* | 30,695,486 | 30,710,510 | 0.052 | [13-17](#_ENREF_13) |
| T1D | *IER3* | 30,710,976 | 30,712,331 | 0.405 | [13-17](#_ENREF_13) |
| T1D | *HCG20* | 30,734,602 | 30,760,027 | 0.338 | [13-17](#_ENREF_13) |
| T1D | *DDR1* | 30,844,198 | 30,867,933 | 0.217 | [13-17](#_ENREF_13) |
| T1D | *VARS2* | 30,876,019 | 30,894,236 | 0.195 | [13-17](#_ENREF_13) |
| T1D | *CCHCR1* | 31,110,216 | 31,126,015 | 0.332 | [13-17](#_ENREF_13) |
| T1D | *HLA-C* | 31,236,526 | 31,239,907 | 0.677 | [13-17](#_ENREF_13) |
| T1D | *MICA* | 31,367,561 | 31,384,016 | 0.157 | [13-17](#_ENREF_13) |
| T1D | *MICB* | 31,462,658 | 31,478,901 | 0.620 | [13-17](#_ENREF_13) |
| T1D | *ATP6V1G2-DDX39B* | 31,497,996 | 31,514,385 | 0.116 | [13-17](#_ENREF_13) |
| T1D | *DDX39B* | 31,497,996 | 31,510,225 | 0.134 | [13-17](#_ENREF_13) |
| T1D | *TNF* | 31,543,344 | 31,546,113 | 0.029 | [13-17](#_ENREF_13) |
| T1D | *LTB* | 31,548,302 | 31,550,299 | 0.050 | [13-17](#_ENREF_13) |
| T1D | *LST1* | 31,553,901 | 31,556,686 | 0.377 | [13-17](#_ENREF_13) |
| T1D | *NCR3* | 31,556,672 | 31,560,762 | 0.032 | [13-17](#_ENREF_13) |
| T1D | *APOM* | 31,620,193 | 31,625,987 | 0.091 | [13-17](#_ENREF_13) |
| T1D | *GPANK1* | 31,629,006 | 31,634,060 | 0.099 | [13-17](#_ENREF_13) |
| T1D | *CSNK2B* | 31,633,013 | 31,638,120 | 0.035 | [13-17](#_ENREF_13) |
| T1D | *LY6G5C* | 31,644,461 | 31,651,817 | 0.248 | [13-17](#_ENREF_13) |
| T1D | *ABHD16A* | 31,654,726 | 31,671,221 | 0.029 | [13-17](#_ENREF_13) |
| T1D | *SAPCD1* | 31,730,576 | 31,732,628 | 0.359 | [13-17](#_ENREF_13) |
| T1D | *LSM2* | 31,765,173 | 31,774,761 | 0.088 | [13-17](#_ENREF_13) |
| T1D | *HSPA1L* | 31,777,396 | 31,783,437 | 0.150 | [13-17](#_ENREF_13) |
| T1D | *HSPA1B* | 31,795,512 | 31,798,031 | 0.028 | [13-17](#_ENREF_13) |
| T1D | *C6orf48* | 31,802,385 | 31,807,541 | 0.020 | [13-17](#_ENREF_13) |
| T1D | *EHMT2* | 31,847,536 | 31,865,464 | 0.072 | [13-17](#_ENREF_13) |
| T1D | *SKIV2L* | 31,926,857 | 31,937,532 | 0.069 | [13-17](#_ENREF_13) |
| T1D | *DOM3Z* | 31,937,587 | 31,940,069 | 0.270 | [13-17](#_ENREF_13) |
| T1D | *ATF6B* | 32,065,907 | 32,096,030 | 0.047 | [17](#_ENREF_17) |
| T1D | *FKBPL* | 32,096,484 | 32,098,068 | 0.022 | [17](#_ENREF_17) |
| T1D | *PRRT1* | 32,116,136 | 32,122,150 | 0.022 | [17](#_ENREF_17) |
| T1D | *XXbac-BPG300A18.12* | 32,121,622 | 32,139,755 | 0.064 | [17](#_ENREF_17) |
| T1D | *AGPAT1* | 32,135,989 | 32,145,873 | 0.046 | [17](#_ENREF_17) |
| T1D | *NOTCH4* | 32,162,620 | 32,191,844 | 0.055 | [17](#_ENREF_17) |
| T1D | *BTNL2* | 32,359,241 | 32,379,511 | 0.035 | [17](#_ENREF_17) |
| T1D | *HLA-DRB1* | 32,546,546 | 32,557,625 | 0.533 | [17](#_ENREF_17) |
| T1D | *HLA-DQA1* | 32,595,956 | 32,614,839 | 0.652 | [17](#_ENREF_17) |
| T1D | *HLA-DQB1* | 32,627,244 | 32,636,160 | 0.821 | [17](#_ENREF_17) |
| T1D | *TAP2* | 32,781,544 | 32,806,599 | 0.815 | [17](#_ENREF_17) |
| T1D | *XXbac-BPG246D15.9* | 32,781,544 | 32,806,599 | 0.137 | [17](#_ENREF_17) |
| T1D | *TAP1* | 32,812,986 | 32,821,755 | 0.066 | [17](#_ENREF_17) |
| T1D | *HLA-DPA1* | 33,032,346 | 33,048,552 | 0.423 |  |
| T1D | *HSD17B8* | 33,172,419 | 33,174,608 | 0.194 |  |
| T1D | *RING1* | 33,176,272 | 33,180,499 | 0.417 |  |
| T1D | *B3GALT4* | 33,244,917 | 33,246,602 | 0.282 |  |
| T1D | *TAPBP* | 33,267,471 | 33,282,164 | 0.206 |  |
|  |  |  |  |  |  |
| RA | *TUBB* | 30,687,978 | 30,693,203 | 0.013 |  |
| RA | *IER3* | 30,710,976 | 30,712,331 | 0.405 |  |
| RA | *HCG20* | 30,734,602 | 30,760,027 | 0.338 |  |
| RA | *DDR1* | 30,844,198 | 30,867,933 | 0.217 |  |
| RA | *HSPA1A* | 31,783,291 | 31,785,723 | 0.029 |  |
| RA | *C6orf48* | 31,802,385 | 31,807,541 | 0.020 |  |
| RA | *NEU1* | 31,825,436 | 31,830,683 | 0.081 |  |
| RA | *DOM3Z* | 31,937,587 | 31,940,069 | 0.270 |  |
| RA | *XXbac-BPG300A18.12* | 32,121,622 | 32,139,755 | 0.064 |  |
| RA | *AGPAT1* | 32,135,989 | 32,145,873 | 0.046 |  |
| RA | *NOTCH4* | 32,162,620 | 32,191,844 | 0.055 |  |
| RA | *HLA-DRB5* | 32,485,120 | 32,498,064 | 0.741 |  |
| RA | *HLA-DQA1* | 32,595,956 | 32,614,839 | 0.652 |  |
| RA | *HLA-DQB1* | 32,627,244 | 32,636,160 | 0.821 |  |
| RA | *XXbac-BPG246D15.9* | 32,781,544 | 32,806,599 | 0.137 |  |
| RA | *HLA-DOA* | 32,971,955 | 32,977,389 | 0.152 |  |
| RA | *TAPBP* | 33,267,471 | 33,282,164 | 0.206 |  |
| RA | *BAK1* | 33,540,324 | 33,548,070 | 0.618 |  |
| RA | *MNF1* | 33,665,345 | 33,679,504 | 0.198 |  |

**Note**: MHC: major histocompatibility complex; T1D: type 1 diabetes; RA: rheumatoid arthritis; *h*^2^ is the estimated heritability value of the gene in the Geuvadis data.

**Reference**

1. Harville, D. A. Bayesian inference for variance components using only error contrasts. *Biometrika* **61**, 383-385, doi:10.1093/biomet/61.2.383 (1974).
2. Patterson, H. D. & Thompson, R. Recovery of interblock information when block sizes are unqual. *Biometrika* **58**, 545-555, doi:10.2307/2334389 (1971).
3. Zeng, P. *et al.* Likelihood Ratio Tests in Rare Variant Detection for Continuous Phenotypes. *Annals of Human Genetics* **78**, 320-332, doi:10.1111/ahg.12071 (2014).
4. Crainiceanu, C. M. & Ruppert, D. Likelihood ratio tests for goodness-of-fit of a nonlinear regression model. *Journal of Multivariate Analysis* **91**, 35-52, doi:10.1016/j.jmva.2004.04.008 (2004).
5. Crainiceanu, C. M. & Ruppert, D. Likelihood ratio tests in linear mixed models with one variance component. *Journal of the Royal Statistical Society: Series B (Statistical Methodology)* **66**, 165-185, doi:10.1111/j.1467-9868.2004.00438.x (2004).
6. Crainiceanu, C., Ruppert, D., Claeskens, G. & Wand, M. P. Exact likelihood ratio tests for penalised splines. *Biometrika* **92**, 91-103, doi:10.1093/biomet/92.1.91 (2005).
7. Scheipl, F., Greven, S. & Küchenhoff, H. Size and power of tests for a zero random effect variance or polynomial regression in additive and linear mixed models. *Computational Statistics & Data Analysis* **52**, 3283-3299, doi:10.1016/j.csda.2007.10.022 (2008).
8. Pinheiro, J. C. & Bates, D. *Mixed-Effects Models in S and S-PLUS*. 2nd edn, (Springer, 2009).
9. Lippert, C. *et al.* Greater power and computational efficiency for kernel-based association testing of sets of genetic variants. *Bioinformatics* **30**, 3206-3214, doi:10.1093/bioinformatics/btu504 (2014).
10. Greven, S., Crainiceanu, C. M., Küchenhoff, H. & Peters, A. Restricted Likelihood Ratio Testing for Zero Variance Components in Linear Mixed Models. *Journal of Computational and Graphical Statistics* **17**, 870-891, doi:10.1198/106186008x386599 (2008).
11. Davies, R. B. Algorithm AS 155: The Distribution of a Linear Combination of chi-2 Random Variables. *Journal of the Royal Statistical Society: Series C (Applied Statistics)* **29**, 323-333, doi:10.2307/2346911 (1980).
12. DIAbetes Genetics Replication And Meta-analysis (DIAGRAM) Consortium *et al.* Genome-wide trans-ancestry meta-analysis provides insight into the genetic architecture of type 2 diabetes susceptibility. *Nature Genetics* **46**, 234-244, doi:10.1038/ng.2897 (2014).
13. Barrett, J. C. *et al.* Genome-wide association study and meta-analysis find that over 40 loci affect risk of type 1 diabetes. *Nature Genetics* **41**, 703-707, doi:10.1038/ng.381 (2009).
14. Cooper, J. D. *et al.* Meta-analysis of genome-wide association study data identifies additional type 1 diabetes risk loci. *Nature Genetics* **40**, 1399-1401, doi:10.1038/ng.249 (2008).
15. The Wellcome Trust Case Control Consortium. Genome-wide association study of 14,000 cases of seven common diseases and 3,000 shared controls. *Nature* **447**, 661-678, doi:10.1038/nature05911 (2007).
16. Hakonarson, H. *et al.* A genome-wide association study identifies KIAA0350 as a type 1 diabetes gene. *Nature* **448**, 591-594, doi:10.1038/nature06010 (2007).
17. Perry, J. R. *et al.* Stratifying type 2 diabetes cases by BMI identifies genetic risk variants in LAMA1 and enrichment for risk variants in lean compared to obese cases. *PLoS Genetics* **8**, e1002741, doi:10.1371/journal.pgen.1002741 (2012).
18. Lin, H. *et al.* Novel susceptibility genes associated with diabetic cataract in a Taiwanese population. *Ophthalmic Genetics* **34**, 35-42, doi:10.3109/13816810.2012.736590 (2013).
19. Nakajima, M. *et al.* New sequence variants in HLA class II/III region associated with susceptibility to knee osteoarthritis identified by genome-wide association study. *PLoS ONE* **5**, e9723, doi:10.1371/journal.pone.0009723 (2010).
20. Julia, A. *et al.* Genome‐wide association study of rheumatoid arthritis in the Spanish population: KLF12 as a risk locus for rheumatoid arthritis susceptibility. *Arthritis and Rheumatism* **58**, 2275-2286, doi:10.1002/art.23623 (2008).
